# Practical Notes on Remedies

**Published:** 1906-06-16

**Authors:** 


### PRACTICAL NOTES ON REMEDIES.

### On the Use of Bichromate of Potash in Dyspepsia.

SOME years ago the use of bichromate of potash in cases of dyspepsia was revived by Frazer, of Edinburgh. A somewhat extensive use of this remedy has shown that in certain conditions, which can easily be defined, it acts most admirably. On the other hand, it only tends to make matters worse if they are not fulfilled. These conditions are a fairly good appetite, a clean tongue, a sense of weight in the stomach with flatulence after food, and, frequently, "water-brash"— *i.e.* regurgitation of an acid-tasting fluid from the stomach. In the particular kind of dyspepsia which is marked by those symptoms a cure may generally be expected from the administration of the drug. It should be given in doses of $\frac{1}{12}$th to $\frac{1}{10}$th of a grain dissolved in 1 oz of water about five minutes before each meal. As a rule, no restriction or alteration of the diet is needed. In some cases it may be advisable to recommend abstention from cooked fats, sugar, and green vegetables. If the tongue is coated, if the appetite is lost, or if the taking of food produces actual pain in the stomach, bichromate of potash not only does no good, but may actually make matters worse. The explanation of these actions is probably to be sought in the fact that bichromate of potash is a slight astringent—it acts favourably on an atonic mucous membrane, but deleteriously if there be any gastric catarrh.

### On the Diagnosis of Flint's Murmur.

ONE of the phenomena of aortic regurgitation is a characteristic diastolic murmur generally hiding the second sound and best heard over the left border of the sternum. In addition to this murmur there may, in some cases, be another—also diastolic in time but later heard over the apex and immediately around, crescendo in character, accompanied by a thrill, and immediately followed by the first sound of the heart. This second murmur, often called Flint's murmur, is thus of exactly the same type and time relations as that found in mitral stenosis. And yet post-mortem examination of such cases of aortic regurgitation may show disease of the aortic valve only, the mitral orifice and valve being normal. The explanation usually given of such cases is that the regurgitating current of blood into the left ventricle impinges on the anterior segment of the mitral valve and presses it back so that a functional stenosis of the orifice results; the physical phenomena so produced consequently exactly imitate those of organic mitral stenosis. Another theory which has been put forward is that the regurgitating current of blood makes the anterior segment of the mitral valve vibrate, and the resulting vibrations in the current of blood entering from the left auricle produce the murmur. This theory, however, does not give so good an explanation of the crescendo character of the murmur. Flint's murmur is apt to be a variable phenomenon; it may be present one day and absent the next. The causes of this variation are as yet but little understood. A difficulty in the diagnosis of Flint's murmur is that mitral valve disease causing organic stenosis may co-exist with aortic valve disease. Such cases may present murmurs precisely similar to those heard in aortic valve disease only—an early diastolic murmur best heard along the left border of the sternum and a late diastolic or presystolic murmur, with accompanying thrill, at the apex. A case of this kind was recently under observation. A boy, who had had rheumatic fever four years previously, suffered from gradually increasing heart failure, with its usual physical signs and symptoms. The apex beat was in the sixth space, one and half inch outside the nipple-line, and there was considerable dilatation of the right side of the heart, as was shown by increase of the superficial cardiac dullness to the right of the sternum and by the presence of a tricuspid systolic murmur. In addition there were the two diastolic murmurs mentioned above. At the post-mortem examination organic stenosis of the mitral valve and disease of the aortic valve were found, with dilatation of all the cardiac cavities and tricuspid orifice, and hypertrophy of the cardiac wells. The emptied heart weighed 23 oz., and it contained 8 oz. of blood clot. The question which arose during life was whether the presystolic murmur indicated organic stenosis of the mitral orifice or whether it was Flint's murmur. The fact that it was associated with a thrill was of no assistance, for a Flint's murmur is also accompanied by a thrill. Its constancy, however, and the presence of great dilatation of the right side of the heart, were strong reasons for thinking that the murmur owned an organic cause. Further, the cause of the valve disease was certainly rheumatism; and, as is well known, this poison rarely affects the aortic valve only. As a rule it is the mitral valve only, or the mitral and aortic valves together, which are affected by the rheumatic virus.

These considerations enabled the prediction to be made with a considerable degree of certainty, that both valves were damaged; and as mentioned above this was found to be the case. If a patient be seen only once, and he presents a presystolic murmur in addition to an aortic regurgitant one, it may not be possible to say whether the presystolic murmur is caused by organic or functional mitral stenosis; but if there be a well-marked dilatation of the right side of the heart, and especially if the presystolic murmur and thrill are constant features in the case, the cause is almost certainly organic stenosis of the mitral valve. On the other hand, if the presystolic murmur and thrill are variable, one has to do with the phenomenon first pointed out by Austin Flint.

### Heart Disease and Palpitation.

THIS little girl has morbus cordis with a very dilated heart. Although she has obvious throbbing she does not complain of palpitation. Patients who complain most of palpitation frequently have no organic disease of the heart. I think you may go farther and say when patients with morbus cordis complain of violent palpitation there is often also a nervous factor in the case.—*Dr. Samuel West.*

